# Combined Strategies to Prompt the Biological Reduction of Chlorinated Aliphatic Hydrocarbons: New Sustainable Options for Bioremediation Application

**DOI:** 10.3390/bioengineering8080109

**Published:** 2021-08-03

**Authors:** Marta M. Rossi, Edoardo Dell’Armi, Laura Lorini, Neda Amanat, Marco Zeppilli, Marianna Villano, Marco Petrangeli Papini

**Affiliations:** Department of Chemistry, Sapienza, University of Rome, Piazzale Aldo Moro 5, 00185 Rome, Italy; edoardo.dellarmi@uniroma1.it (E.D.); laura.lorini@uniroma1.it (L.L.); neda.amanat@uniroma1.it (N.A.); marco.zeppilli@uniroma1.it (M.Z.); marianna.villano@uniroma1.it (M.V.); marco.petrangelipapini@uniroma1.it (M.P.P.)

**Keywords:** bioremediation, biological reductive dechlorination, chlorinated aliphatic hydrocarbons, sustainable materials, biochar, polyhydroxyalkanoates, bioelectrochemical systems

## Abstract

Groundwater remediation is one of the main objectives to minimize environmental impacts and health risks. Chlorinated aliphatic hydrocarbons contamination is prevalent and presents particularly challenging scenarios to manage with a single strategy. Different technologies can manage contamination sources and plumes, although they are usually energy-intensive processes. Interesting alternatives involve in-situ bioremediation strategies, which allow the chlorinated contaminant to be converted into non-toxic compounds by indigenous microbial activity. Despite several advantages offered by the bioremediation approaches, some limitations, like the relatively low reaction rates and the difficulty in the management and control of the microbial activity, can affect the effectiveness of a bioremediation approach. However, those issues can be addressed through coupling different strategies to increase the efficiency of the bioremediation strategy. This mini review describes different strategies to induce the reduction dechlorination reaction by the utilization of innovative strategies, which include the increase or the reduction of contaminant mobility as well as the use of innovative strategies of the reductive power supply. Subsequently, three future approaches for a greener and more sustainable intervention are proposed. In particular, two bio-based materials from renewable resources are intended as alternative, long-lasting electron-donor sources (e.g., polyhydroxyalkanoates from mixed microbial cultures) and a low-cost adsorbent (e.g., biochar from bio-waste). Finally, attention is drawn to novel bio-electrochemical systems that use electric current to stimulate biological reactions.

## 1. Background

Chlorinated aliphatic hydrocarbons (CAHs) are ubiquitous contaminants whose presence in groundwater has persisted for many decades, mainly due to the physical-chemical characteristics of these compounds [1,2]. In particular, CAHs belong to Dense Non-Aqueous Phase Liquids (DNAPLs), for which contamination scenarios differ significantly from Light NAPLs scenarios, where the separate phase floats at the top of the water table due to its lower density than water [3,4].

The two major problems associated with aged CAHs-contaminated sites are the secondary source management and the containment and remediation of the contamination plume. Secondary source refers to the presence of the solvent in a separate phase, primarily accumulated in the low-permeability layers of the aquifer (clay lens), which work as a source of long-term contaminants [5]. On the other hand, contamination plume refers to prolonged contamination in the same direction of water flow due to the slow and steady release of secondary sources. The distribution of contaminants is highly dependent on the hydrogeological characteristics of the site and concentration levels differing greatly from source to source.

**Figure 1:**
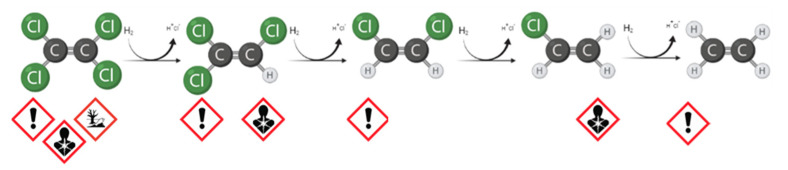
Step-by-step reductive dechlorination (RD) and hazard pictograms associated with each compound. Created with Biorender.com (accessed on 1 April 2021).

Today, in-situ technologies can operate and significantly reduce costs, although detailed site characterization is required. In this context, many improvements have been made in modeling and interpreting the data to support the design and adoption of the right strategy [6]. For the remediation of chlorinated solvents plume, the In-Situ Bioremediation (ISB) technologies are growing attention. The reasons for the success are the public support, success on the dissolved contaminants, and a comparatively low cost when it is effective [7]. If the presence of degradation intermediates is detected during site characterization, the biological Natural Attenuation phenomenon occurs due to the presence of specific microorganisms [8]. For the reductive dechlorination (RD) of widespread contaminants, such as tetrachloroethane (TeCA), perchloroethylene (PCE), and trichloroethylene (TCE), in an anaerobic environment, one of the most widely used approaches is enhanced natural attenuation (ENA) [9,10]. The intervention provides the addition of an organic fermentable substrate to produce short-chain fatty acids (acetate and other volatile fatty acids, VFAs) and the direct electron donor, the molecular hydrogen [11,12]. Therefore, almost any fermentable substrate can be a potential source of carbon and hydrogen to stimulate RD, including carbohydrates (sugars), alcohols, oils, solids (e.g., bark mulch, chitin), and complex compounds (e.g., whey and cellulose) [13]. The RD is a well-known, step-by-step reaction. Focusing on the sequential RD pathway of PCE through TCE, cis-dichloroethyene (cis-DCE), and vinil chloride (VC) to ethene, shown in Figure 1, the efficiency of each step can be dramatically different depending on the environmental conditions and the microbial populations responsible for the reactions [14,15]. If the specific population has developed in the aquifer (*Dehalococcoides* spp.), the reaction may proceed until ethene production. As with every technology, serious issues may be associated with ISB, and the following situation may seriously affect meeting the remediation goals:

- Low efficacy on separate phase or highly adsorbed fraction (at source);
- Substrate availability limitations; and
- Incomplete degradation pathways (stall at an intermediate stage) for energetic and kinetic constraints with volatile and more toxic by-product accumulation, e.g., VC.

Recently, the combined approach has been investigated to help address the RD limits. Complete management of the site can be achieved more effectively with a combination of physicochemical processes to prompt anaerobic biodegradation [16]. For example, over the last few decades, the feasibility of DNAPLs’ bioremediation, also in the source areas, has been recognized through the combination of ISB with other more aggressive technologies, such as ISCO-R (In-Situ Chemical Oxidation Reduction), thermal treatment, and excavation [17]. In addition, the coupled technology is expected to be more satisfactory than a single-technology approach to deal with site changes and response during the intervention. Compared to individual methods, coupling technologies into sequences, combinations, or treatment trains can be more effective in reducing the contamination level and in the overall duration. As concerning the specific RD reactions, it is well known that the anaerobic condition favors the degradation of the highly chlorinated compounds, whereas aerobic condition favors the oxidation of di- and mono-substituted compounds [14]. For this reason, the sequential combination of technologies that might be considered opposing (reduction/oxidation) may be more effective to avoid VC accumulation. In this work, we described field and research studies of the last years that report effective and innovative interventions to enhance the RD in secondary sources and plume of CAHs contamination. Intervention strategies are described where the coupling of chemical/physical processes succeeded in the stimulation of dechlorinating microorganisms present on the site. The review then moved to other bio-based materials (i.e., biochar from agricultural wastes and polyhydroxyalkanoates (PHA) from mixed microbial culture) and new microbial electrochemical technologies (MET) configurations to better meet the goals of the circular economy.

## 2. Coupled Strategies: Case Studies

### 2.1. Combination of GCW (Groundwater Circulation Well) and ENA (Enhanced Natural Attenuation)

To gain the remediation of an aged source zone of CAHs, Petrangeli Papini and co-workers (2016) performed a pilot test in Italy in a complex operative industrial site contaminated by different chlorinated solvents (at a concentration up to 100 mg/L) [18,19]. Groundwater circulation wells (GCWs) (30 m deep) were designed to create the groundwater circulation through the treatment zone, both horizontally and vertically, and contemporary amendment addition was realized to enhance the ISB. GCW is a novel approach for effective mobilization of contaminants for the secondary source and to provide optimal conditions for better contact between microbes, contaminants, amendments, and nutrients distribution [20].

**Figure 2:**
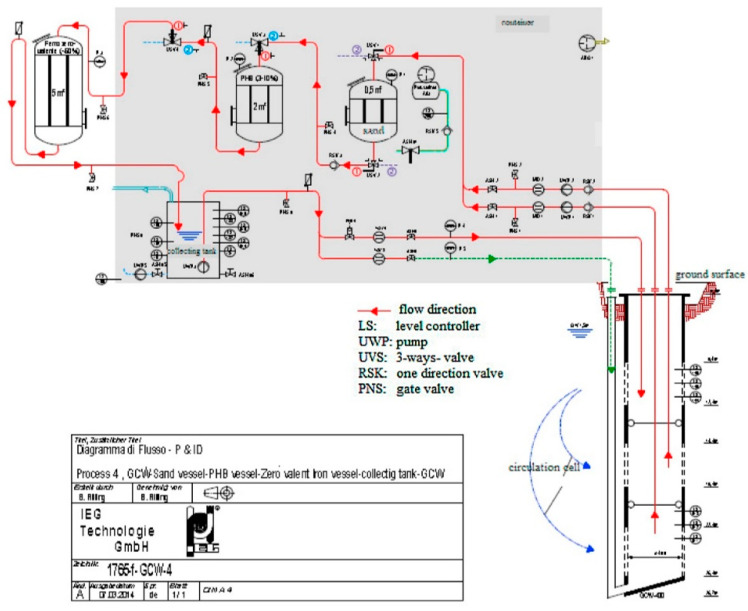
Piping and instrumentation diagram of the groundwater circulation well (GCW) and the polyhydroxybutyrate (PHB)-reactor and zero-valent iron (ZVI)/PHB-reactor in series. Figure from Pierro et al., 2017 [21]. Reproduced with the permission of Elsevier.

Previous characterization of the site and microcosms tests assessed the possibility to significantly enhance the biodegradative potential of natural microbiota with the addition of electron donors [19]. Because of GCW, the low-permeable layer was constantly penetrated by the vertical flow, allowing the mobilization of the residual phase. The extracted water from GCW flowed to the external treatment unit composed of sand and biodegradable polymer (polyhydroxybutyrate, PHB) reactor (where fermentation takes place) and a zero-valent iron (ZVI) reactor before reinjection (Figure 2). In this case, a slow-release electron donor source, the PHB, was also investigated. Results from laboratory tests to pilot-scale demonstrated the efficiency of the coupled strategy, enhancing the mobilization of CAHs and stimulating the biological processes [21]. The mass of contaminants removed during the eight-month monitoring was comparable with a traditional pump-and-treat method, but the overall processing time is still difficult to estimate [18]. Additionally, microbial analysis performed by Matturro and colleagues (2018) reported that the slow-release source of electron donors in the field allowed the establishment of a stable population of *Dehalococcoides mccartyi*, mainly carrying *bvcA* and *vcrA* genes (encoding for the reductive dehalogenase BvcA and VcrA, respectively), which are essential to complete the RD steps to harmless ethene [22,23]. As reported by O’Connor et al. (2018) the interest in Controlled Release Materials (CRM) is increased in the environmental remediation field [24]; hence, PHB can be included in these materials as a controlled H_2_-release. Aulenta et al. (2008) and Baric and coauthors (2012) already studied and investigated the fermentability of this bio-polymer and its possible use for enhancing the performance of ZVI towards chlorinated ethane, increasing iron reactivity and longevity, and enhancing biological reductive processes [25,26].

### 2.2. Combination of Adsorption and Biodegradation

The first example in Europe of completed remediation of an aquifer contaminated with CAHs by a combination of adsorption and biodegradation was reported by Ciampi and colleagues (2019) [27]. In detail, this work highlighted the importance of the integrated use of geological, hydrogeological, chemical, and geophysical data to achieve high-resolution characterization of the site (the new high-speed railway station of Bologna, Italy). The concentration levels of PCE were between 1.1–110 μg/L and those of TCE in the range of 1.5–150 μg/L, even exceeding the Italian threshold limits (CSC) by an order of magnitude for each contaminant (CSC are 1.1 and 1.5 μg/L for PCE and TCE, respectively). A commercial colloidal activated carbon, PlumeStop™ (Regenesis, San Clemente, CA, USA), was co-injected with an electron donor (HRC™, Regenesis, San Clemente, CA, USA) to provide bio-stimulation. The colloidal-activated carbon product, essentially a micrometric-activated carbon superficially modified for better distribution, created an in-situ adsorption zone. An efficient response was immediate, indeed; after the first weeks of injection, the reduction in concentration was evident, and levels of contaminants were stable over time. If the traditional activated carbon treatment only results in the transfer of contaminants from liquid or gaseous streams onto the adsorbent material without actual degradation into harmless final products, the simultaneous adsorption and biodegradation strategy should also overcome adsorbent material saturation, prolonging the operational lifespan of the reactive media and the kinetic limitation of RD [28]. The use of activated carbon as a support for PCE degradation was already investigated by Yunhai Wu et al. (2000), who described how PCE was microbially bio-transformed and adsorbed on granular biological activated carbon (GBAC) [29]. Other authors are studying this process on different types of contaminants [30]. The biofilm-GAC filters for micropollutants degradation were recently published by Piai and co-authors (2020) [31]. The study demonstrated that sorption and biodegradation occur simultaneously, leading to an efficient bio-regeneration of the GAC [31]. These promising results open doors to different configurations of application, such as the direct injection of the reactive adsorption products in the aquifer or the installation of a bio-barrier. Alternatively, an external bioreactor packed with a mixture of adsorption material/electron donor may also be feasible for the extracted water’s treatment.

## 3. Novel Research and Perspectives

### 3.1. Sustainable Electron Donor Source: PHA from Mixed Microbial Culture

The implementation of enhanced bioremediation technology calls for the development of systems for the distribution of suitable electron-donor compounds. In general, the addition is carried out by direct push/injection wells, by the construction of bio-walls, or by injection/extraction wells to recirculate and improve the distribution of the amendments [32]. For the success of biological metabolism, the use of a slow-release, low-solubility substrate for hydrogen donation should be a valuable approach (e.g., HRC™ or vegetable oils) [33]. When injected into the aquifer, these substrates slowly hydrolyze to form more soluble compounds that are transported along the contamination plume. Although H_2_ is rapidly consumed by several consortia of bacteria in the subsurface, VFAs (e.g., acetate) migrate down into a larger contaminated area. For this reason, long-lived, controlled release substrates are preferable to short-term, rapidly degraded sources of carbon. In addition, the benefits of long-lived substrates also include the need for less frequent injections, thereby reducing operational requirements, thus reducing costs [32].

**Figure 3:**
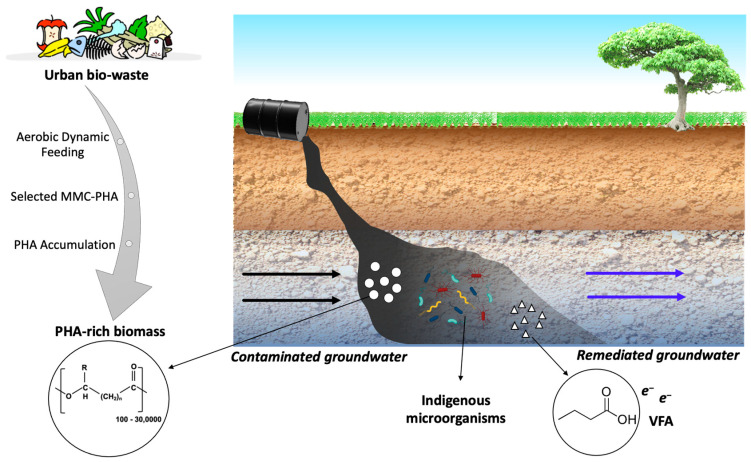
Schematic application of polyhydroxyalkanoates (PHA) from selected mixed microbial culture (MMC) for biological reductive dechlorination (RD). Created with Biorender.com (accessed on 1 April 2021).

As mentioned above, the use of PHB and other polyesters open doors to a possible configuration to improve RD. Examples include the construction of reactive permeable biological barriers using PHB and ZVI or engineered composite materials. A newly developed nano-ZVI/PHB composite material was synthesized by Chronopolou et al. (2016) based on the interaction of iron particles (the core) and the bio-polymer (PHB, the surrounding shell) [34]. The positive effect of ISCR with ZVI and biostimulation to clean up the CAHs source area has been shown by both a microcosm experiment [35] and a field application. Wu et al. (2020) reported a removal percentage of 97.5% of chlorinated ethylene and 80.2% of chlorinated ethane after 253 days post injection (slurry made with micro-ZVI and biostimulant components) [36]. To better fit a circular economy approach, a more sustainable option should be considered through the use of polyhydroxyalkanoates (PHA) produced from renewable resources. PHA means a large group of homopolymers and copolymers in the class of polyesters, including PHB. They are all biologically derived and biocompatible and biodegradable in the environment by many ubiquitous microorganisms [37]. Typically, commercial PHB is produced by pure cultivation under straighter conditions, resulting in higher production costs (2.0–5.0 €/kg) [38]. Nowadays, new technology to produce cost-effective PHA is well developed, using mixed microbial cultures (MMCs) [39]. This strategy also makes it possible to combine wastewater treatment with bio-plastic production [40,41]. Indeed, PHA-accumulating organisms can be selected from typical activated sludge from wastewater treatment plants using Aerobic Dynamic Feeding (ADF) conditions [42]. Lorini and colleagues (2021) pointed out the possibility of exploiting various organic biowaste as a raw material for the production of purified PHA with properties comparable to those of commercial plastics [43]. The investigated PHA were produced in three pilot platforms: in Treviso (Northeast of Italy) [41], Carbonera (Northeast of Italy) [44], and Lisbon. Each process was based on the selection and enrichment of PHA-producing biomass from MMCs and the exploitation of different fermentable organic waste (i.e., a mixture of the organic fraction of municipal solid waste and sewage sludge; cellulosic primary sludge; fruit waste, respectively) [43]. Recently, our research group examined the fermentability of these innovative PHA materials in comparison with commercial ones. Amanat et al. (2021) studied the MMC-based materials produced in the previously mentioned pilot unit in Treviso (Italy) and Lisbon (Portugal) as well as PHB available on the market [45]. As a major finding, a much shorter start to the fermentation process was necessary for the MMC raw-PHA material. This material was composed of PHA-rich biomass coming from the accumulation stage of the MMC-PHA production process without undergoing extraction and purification procedures. The results suggest that it can be used directly as a sustainable and effective carbon source with remarkable economic and environmental benefits (Figure 3). Furthermore, the whole set of materials was also analyzed for the determination of relevant contaminants, such as heavy metals [46], polycyclic aromatic hydrocarbons (PAH) [47], and polychlorinated biphenyls (PCB) [48]. The studies demonstrated that all the MMC-PHAs met regulatory standards required for traditional plastic materials. Therefore, the use of organic waste as feedstock for PHA production processes appears to be safe for the environment and human health.

### 3.2. Sustainable Adsorption Material: Biochar

**Figure 4:**
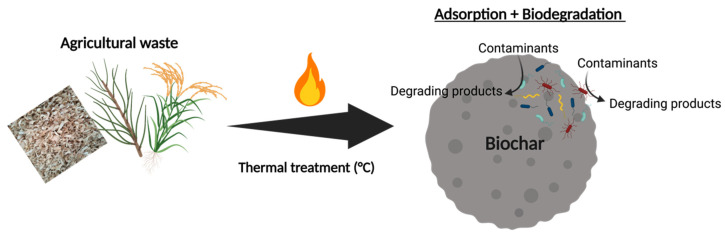
From agriculture bio-waste to selective biofilm supporting material coupling adsorption and contaminants biodegradation. Created with Biorender.com (accessed on 1 April 2021).

The use of secondary material resources is an urgent area for solving energy recovery and for the protection of the environment. Pyrolysis or gasification of waste, particularly biomass from agricultural residues, is a potential resource economy approach [49]. Pyrolysis processes have been continuously improved and are widely used in the manufacture of coke and charcoal. Today, the scientific community has concentrated its attention on the solid residues of pyrolysis or gasification of biomass, known as biochar. With the abundance of raw materials from the massive production of agricultural waste worldwide, biochar has always been recognized as a low-cost product. The production and availability of biochar seem feasible and the routines promising. This is clearly shown by the exponential growth in the number of publications on biochar [50,51]. Moreover, biochar is seen as a new tool to establish environmental management and sustainable energy production. The biochar matrix is similar to an activated carbon, although the commercial one has a higher microporosity and surface area due to the activation process [52]. As a result of no secondary activation process, biochar should be an impressive economic contaminant adsorbent that can be developed from energy recovering with a beneficial role in water treatment, greenhouse gas mitigation, and soil enhancement [53]. The variability of physio-chemical properties enables biochar to optimize its efficiency in targeted applications [54]. While biochar has been used by humans for centuries as a soil supplement, the material has now been acknowledged for its adsorptive properties [55]. Wu Ping and colleagues (2019) published a detailed review of the works of literature concerning biochar between 1998 and 2018 [56]. China is the region with the largest number of publications, evidently because the region’s agricultural technologies are the foremost in the world. Especially during 2011–2015 and 2016–2018, the most used keywords related to biochar were “sorption”, “activated carbon”, “heavy metals”, “pyrolysis”, and “removal”. During this period, the literature describing biochar properties and their application for contaminant immobilization increased [57]. For instance, biochar has recently been studied as a sorbent material to remove metals and organic compounds from aqueous solutions [52,58]. Recently, biochars from different raw materials were compared in terms of the adsorption capacity of TCE (expressed as mg of TCE adsorbed per gram of material) determined by a batch assay [59]. Because of textural characteristics, it is also demonstrated that biochar has a positive effect on improving soil quality, such as increasing total specific soil area, enhancing soil structure and aeration, and creating good habitats for microorganisms [60,61]. Biochar’s macropores may provide appropriate dimensions for bacteria (microbial cells generally measure between 0.5 and 5 µm) [61,62]. Furthermore, some authors have considered the potential role of biochar as an electron acceptor for microbial extracellular respiration and growth [63,64,65]. The research conducted by Cruz Viggi and colleagues (2017) [66] confirmed the importance of electrochemical properties in the biochar-bacteria relationship. Three biochars were tested for their capacity to enhance methane production from a food-waste fermentate. The study suggested that a simple electrochemical test for the determination of electron donation capacity (EDC) might be sufficient to select the most appropriate biochar for anaerobic digestion [66]. Because of these interesting results, recent studies point to biochar as possible support for the growth of specific bacterial consortia and the enhancing of biodegradation of pollutants (Figure 4) [67]. The purpose of this interest is to insert a low-cost material already approved as a fertilizer in the context of environmental matrix re-qualification.

The newest study on the simultaneous adsorption and biodegradation process on two biochars was published by Siggins et al. (2021), in which the development of a TCE dechlorination biofilm was studied. The main goal was to find an alternative adsorption material that could be used in permeable reactive barriers (PRBs) to treat the source of plumes contaminated by TCE and extend the system’s operational life. Both biochars-columns (from herbal pomace and spruce biochar) reported a TCE removal ≥ of 99.7%, and also cis-1,2 DCE was detected adsorbed to biochar [28]. Other studies are reported in support of the good coupled strategy by using recycled material such as biochar but mainly have different targets of contaminated compounds [68,69,70,71]. Further investigations are needed concerning chlorinated solvents and RD supported by biochar, especially to evaluate if this low-cost material may support a strictly selected dechlorinating biofilm (mainly enriched in *Dehalococcoides*). Furthermore, it becomes important to investigate the specific interaction between biochar and daughter products (particularly VC) since they may lose affinity for the material due to an increase in polarity/ hydrophilicity. At the same time, promoting the development of biochar guidelines or quality standards is a critical issue but essential for its safe application. As suggested by the Hu Qiang Group (2020) to promote global biochar-based circular economy technology, other investigations are urgently needed [72]. Sharing information and collaboration with biochar producers is essential, combined with a control mechanism to certify the biochar before environmental application, i.e., for field application to support ISB.

### 3.3. New Configurations of Microbial Electrochemical Technologies (MET)

As referred to in the opening paragraph, microbial RD is a step-by-step reaction. For each stage, one chlorine atom is lost and replaced with one hydrogen atom (by oxidation of H_2_). The improved RD is typically obtained by injecting chemicals into the contaminated matrix. Whereas slow-release substrates can improve yield, the addition of organic matter to the aquifer can also have potential disadvantages [73]. As an example, high dissolved organic matter content can result in deteriorating water quality, metal mobilization, and gas production (e.g., CH_4_). Moreover, the rates and efficacy of RD reactions should not be controlled due to the non-selectivity of the ongoing reaction. For instance, MET are an innovative strategy to control the microorganism’s metabolism through the utilization of simple electrochemical devices [74,75].

**Figure 5:**
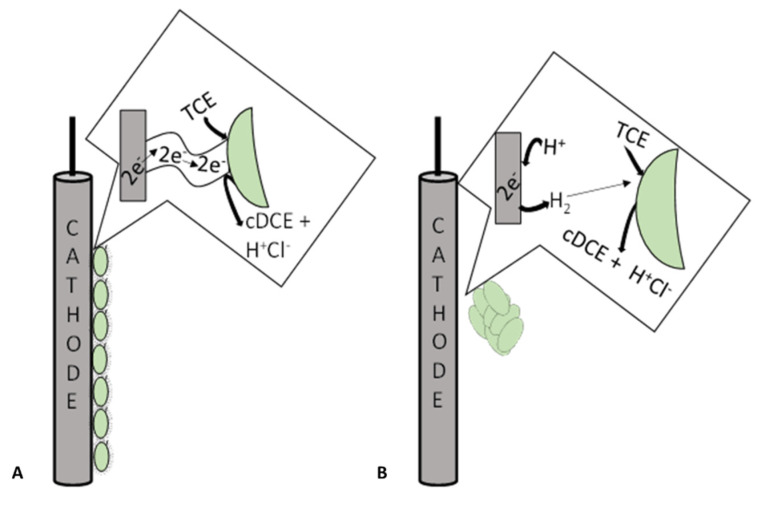
Schematic representation of dechlorinating microorganisms’ activity through direct (**A**) or indirect (**B**) mechanisms on the biocathode.

MET are innovative technologies that exploit the capability of microorganisms to interact with conductive materials to exchange electrons through the mechanism of extracellular electron transfer (EET) [76]. The EET mechanisms can be performed directly by the presence of specialized membrane proteins and their extensions or indirectly through the utilization of redox mediators, which have the function of electron shuttles (Figure 5). Usually, electroactive microorganisms organize themselves in a biofilm on the electrode surface [77,78]. The biofilm uses the conductive material as an electron acceptor of the metabolism; thus, the bio-electrochemical interface is named bioanode. On the contrary, in a biocathode, the conductive material acts as an electron donor of the microbial metabolism. MET can be divided into two main device typologies represented by the microbial fuel cell (MFC) and the microbial electrolysis cell (MEC). MFC constitutes the energetic application of the MET and is substantially based on the electricity production through the combination of the oxidation of organic substrates by a bioanode combined with an oxygen-reducing cathode. MEC requires the application of external energy input for the overcome of the thermodynamic and kinetic barrier of non-spontaneous reactions.

MECs have been explored for several bioremediation applications, including CAHs removal through the RD stimulation. Indeed, the use of a biocathode allows for the supply of reducing power to the dechlorinating microorganisms through direct or indirect mechanisms.

Aulenta and his colleagues (2007) explored the capacity of a carbon solid-state electrode, polarized at −500 mV vs. SHE (Standard Hydrogen Electrode) and coupled with a low-potential redox mediator (methyl-violagen), to sustain RD of TCE to unharmful by-products [73]. This first experimentation garnered interest to investigate the role of H_2_ evolution on the cathode and the possibility to control the rate and the selectivity of the RD [79]. Nowadays, the direct interaction has not yet been proved for dechlorinating-reducing bacteria, and most dedicated studies should be conducted to evaluate the role of hydrogen evolution at the cathode and if this represents the most common reducing power-transfer mechanisms, which, in turn, represents an efficient strategy to supply micronized bubbles of H_2_ directly to the biofilm, avoiding mass transfer limitation, which affects conventional hydrogenophilic approaches [74,76].

Nevertheless, Aulenta et al. (2009) suggested that some endogenous redox mediators can be produced and secreted in response to electrode polarization [80]. A recently published example of a possible in-situ configuration MET is the bioelectric well realized by Palma et al. (2017), where a bioelectrochemical reactor was realized to be inserted directly within groundwater wells (in the study, a petroleum hydrocarbon contamination was investigated) [81].

**Figure 6:**
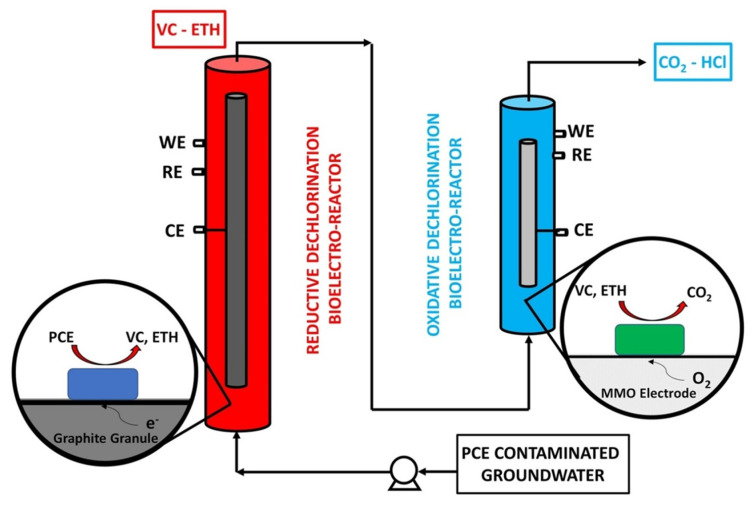
Reductive/oxidative bioelectrochemical reactors combined for biodegradation of PCE and daughter products. Reproduced with the permission of Elsevier, 2020 [87].

On the elimination of chlorinated compounds, a long-term study was conducted. A bioelectrochemical reactor operated uninterrupted for more than 470 days, with stable and reproducible performance in removing TCE without an organic carbon source [82]. Various BES configurations have been tested more recently, looking at sequential coupled strategy and reductive and oxidative dechlorination reactions, respectively, on cathodes and anodes. As mentioned earlier, the oxidation of less-chlorinated intermediates can be energetically favored. Sequential reductive-oxidative treatment was developed by Lai and colleagues (2016) to address the bioremediation of groundwater contaminated by TCE and daughter products, such as VC. In detail, the intermediates were formed in the anaerobic cathodic chamber and degraded in the aerobic anodic chamber. Microbial oxidation of TCE intermediates was successfully performed using an electrolytic system that uses commercial Ti anodes with mixed metallic oxide coating [83]. A new cost-cutting approach is carried out through an innovative process configuration in which two MECs are connected in series (reduction reactor first, followed by the oxidative reactor), avoiding the use of an ion-exchanger membrane to separate the chambers. This particular configuration (made for future pilot testing) was developed by Zeppilli et al. (2019) [84] and studied in depth for PCE removal, also assessing the effect of different feeding solutions in overall performance [85,86]. A schematic draw of the experimental setup is shown in Figure 6. A contaminated solution initially entered the reducing reactor. The cathodic chamber supplied the reducing power to PCE-dechlorinating biomass. Subsequently, a titanium electrode activated with metal mixed oxides facilitated aerobic dechlorination of the lower-chlorinated PCE by-products in the second oxidizing reactor. The average PCE-removal efficiencies reached values as high as 100 ± 1% and 97 ± 1% during the operation at −350 and −550 mV vs. SHE, respectively. Positively, 94 ± 1% and 92 ± 2% of the VC produced by the first step was removed during the same runs thanks to the second oxidative step. The significance of the process combination is also demonstrated by microbial analysis, which showed the development of different redox niches in each MEC compartment. Highly concentrated *D. mccartyi* was found in the reduction reactor [87]. The combined systems and the absence of a separation membrane make this BES reactor less complex and more achievable from a scaling perspective. The system has demonstrated over the last two years that it self-adjusts according to input composition without substrate addition for H_2_ supply and VC accumulation.

## 4. Conclusions

This review paper described the integration of different intervention strategies addressed to prompt the dechlorinating activity of microbiological communities. Such interventions were able to act efficiently on secondary contamination sources, on the one hand mobilizing from the residual phases and stimulating biological activity (GCW-ENA), and on the other hand, quickly reducing the spread of the plume of contamination by immobilizing the contaminant with a micrometric adsorption product and stimulating the activity of naturally present microorganisms (adsorption-biodegradation). Thereafter, two alternative, bio-based materials have been described as a possible alternative to adsorption material and electron-donor source. Biochar is proposed as an eco-friendly and sustainable material known also for its positive interaction with subsoil bacteria. PHA from MMC because is a very promising material and represents a concrete possibility to respect the concept of circular economy and valorization of wastes. However, there is still a lack of literature studies involving MMC-PHA in alternative areas, such as the requalification of environmental matrices. Despite the promising results and progress made in the last years, the bioelectrochemical systems are still investigated on the laboratory scale. In addition, these proposed materials and technology configurations can be coupled together to remove this class of contaminants rapidly and effectively.

## Data Availability

Not applicable.
